# A Multibeam Antenna Array with Controllable Gain Allocation

**DOI:** 10.3390/s26206649

**Published:** 2026-10-21

**Authors:** Chenshuo Chu, Haitao Lu, Peng Xu, Xiao Cai

**Affiliations:** 1 Research Center of Applied Electromagnetics, Nanjing University of Information Science and Technology, Nanjing 210044, China; chuchenshuo1998@126.com (C.C.); lu_haitao@126.com (H.L.); 2 School of Information Engineering, Jiangsu Maritime Institute, Nanjing 211199, China; xup2016@126.com

**Keywords:** array synthesis, beamforming, gain control, nine-beam antenna, power transmission efficiency

## Abstract

Multibeam synthesis often relies on iterative optimization, whose computational cost and convergence time can increase substantially when the gains of different beams are unevenly distributed, making direct control of their relative gains difficult. To address this issue, a multibeam array synthesis and gain-allocation method based on the weighted maximum power transfer efficiency formulation is proposed. The method is developed for operation at 5.8 GHz using an 8 × 8 microstrip transmitting array and a 3 × 3 receiving-ports configuration to synthesize one broadside beam and eight off-axis beams distributed in the azimuth plane. Rx-port locations define the beam directions, and a diagonal weighting matrix sets their relative gains. Given the full-port scattering parameters, the complex array excitation comes from one eigenvalue problem. With equal weights, the nine-beam pattern is maintained for off-axis angles of 35°, 45°, and 55°, with peak gains of 13.3, 12.0, and 11.8 dBi, respectively. At an off-axis angle of 45°, varying the weighting matrix adjusts the gain difference between the central beam and the average gain of the eight off-axis beams from 0 dB to +5 dB or −5 dB, while keeping the beam directions unchanged. The proposed method is intuitive to formulate, accounts for mutual coupling through the full-port S-parameters, and allows gain allocation among multiple beams.

## 1. Introduction

Multibeam antennas can form radiation beams in multiple predefined directions and are of significant value in applications such as multiuser communications, radar detection, satellite communications, and regional coverage [1]. As wireless communication systems place increasing demands on spatial reuse and coverage capability, multibeam antennas have been progressively adopted in 3G, LTE, 5G, and beyond-5G mobile communication systems [2]. In broadband and high-capacity communication systems, multibeam arrays must also account for operating bandwidth, polarization, and performance consistency among different beams [3]. For applications such as base-station coverage, large-scale multibeam arrays have also demonstrated considerable potential for practical implementation [4].

Multibeam antennas are commonly implemented with an antenna array and a beamforming network. Single-layer feeding networks have been used to meet planar and integrated implementation requirements [5], and substrate-integrated waveguide (SIW) networks have been used at millimeter-wave frequencies [6]. Increasing the number of input and output ports can provide more available beams [7].

Beyond one-dimensional multibeam generation, two-dimensional spatial scanning has received growing attention. Two-dimensional beamforming networks control beam directions in two orthogonal dimensions [8], whereas planar phase-shifting structures can reduce the thickness and interconnection complexity of the feeding network [9]. Higher spatial gain is generally obtained by enlarging the array aperture and increasing the element count [10], which is relevant to radar and high-gain communication applications. Recent studies have reported dual-band multibeam networks [11], broadband two-dimensional beamforming networks [12], and feeding networks that offer flexible beam angles and low sidelobe levels [13]. Broadband SIW structures have also extended the operating bandwidth of millimeter-wave multibeam antennas [14]. Together, these studies address bandwidth, integration, and two-dimensional coverage.

Array-pattern synthesis is another route to generating prescribed beams. Convex optimization can impose main-lobe, sidelobe, and null specifications directly and solve for the associated array excitations [15]. As the array size and number of design constraints increase, deterministic and stochastic optimization methods have been applied to phased-array pattern synthesis [16]. Error models can be included to account for practical amplitude and phase errors [17]. Neural networks and surrogate models have also been used to reduce the cost of repeated optimization [18].

Despite their flexibility for complex pattern design, these approaches do not provide the same means of setting inter-beam gain allocation. Fixed beamforming networks, such as Butler matrices [19], generate discrete beams in hardware. Their beam count and inter-beam amplitude-phase relationships follow the network’s power distribution; once the hardware is fixed, the relative beam gains are difficult to reconfigure. Iterative approaches, including genetic algorithms [20], convex optimization [21], particle swarm optimization [22], and simulated annealing [23], allow constraints on beam pointing, sidelobe level, and nulls. However, they require repeated iterations, and the relative beam gains usually result from the optimization rather than being prescribed independently before the optimization.

The weighted method of maximum power transmission efficiency (WMMPTE) provides a different approach to this problem. It reformulates array synthesis as a power-transmission problem between transmitting ports and target receiving ports, and determines the array excitation using port transmission parameters [24]. Because the solution is directly based on network parameters, the effects of mutual coupling, port matching, and the actual array structure can be naturally incorporated into the model.

WMMPTE has been applied to problems such as flat-top pattern synthesis and power focusing toward multiple targets [25,26]. For multiple target directions, multiple receiving ports can be introduced correspondingly. When identical weights are assigned to all receiving ports, the solution tends to produce a relatively balanced energy distribution among the target directions. When different port weights are used, the relative contribution of each target direction to the optimization objective can be adjusted. Therefore, a weighting matrix can be incorporated into the WMMPTE framework so that the beam directions and relative beam gains are controlled by the target-port locations and port weights, respectively.

Based on the above considerations, this work investigates the synthesis and gain control of a nine-beam array using WMMPTE and its weighted formulation. The considered configuration consists of one broadside beam normal to the array aperture and eight off-axis beams uniformly distributed in azimuth. First, the nine-beam radiation characteristics are investigated when equal weights are assigned to all target directions. The weight corresponding to the central beam is then varied to study gain redistribution between the central and off-axis beams. Finally, the off-axis target directions are changed to evaluate the multibeam synthesis capability at different off-axis angles. The main objective of this work is to establish an array synthesis method capable of controlling the relative gains among multiple beams.

The core of this method lies in unifying the target direction setting, port transmission relationship, and beam gain allocation into a single model. The target direction is determined by the position of the receive port, the relative gain is adjusted by the receive port weights, and the array excitation is obtained through solving a matrix eigenvalue problem. This approach preserves the clear physical meaning of the beam direction while avoiding the need to redesign the fixed feeding network for each gain allocation.

The rest of the paper is organized as follows. Section 2 presents WMMPTE, including the array-excitation solution derived from the transmission matrix between the transmitting and receiving ports. Section 3 describes the nine-beam array and the target-direction configuration. Section 4 examines the reflection coefficients, equal-gain nine-beam formation, weighted gain control, and off-axis-angle scanning. Section 5 discusses the numerical results, practical implementation, and comparison with published multibeam methods. Section 6 concludes the paper.

## 2. WMMPTE Multibeam Formation Principle

Assume that the Tx array contains *N* transmitting ports, and that the target spatial directions are represented by *M* Rx ports. The transmission matrix from the Tx ports to the Rx ports, denoted as $[S]\in {\mathbb{C}}^{M\times N}$, is obtained by full-wave simulation or measurement. Let [a] be the excitation vector of the Tx ports. Then, the response at the Rx ports can be written as [b]=[S][a]. To adjust the relative importance of different target directions, a Tx-port weighting matrix $[W]=diag\left(w_{0},w_{1},w_{2},\ldots ,w_{8}\right)$ is introduced. According to multiport network theory and the WMMPTE modeling concept, the weighted received power can be expressed as [24,25,26,27,28]

(1) $$P_{rec}={\left[a\right]}^{H}{\left[S\right]}^{H}{\left[W\right]}^{H}[W][S][a]$$

Let

(2) $$\left[A]={\left[S\right]}^{H}{\left[W\right]}^{H}[W][S\right]$$

Under the normalized transmit-power condition ${\left[a\right]}^{H}[a]=1$, the optimal excitation can be obtained from the following eigenvalue problem:

(3) $$\left[A][a_{opt}]=\lambda_{max}\;[a_{opt}\right]$$

The eigenvector $\left[a_{opt}\right]$ corresponding to the maximum eigenvalue is the optimal complex excitation of the Tx array. The power weight and phase of the nth Tx port are, respectively, given by

(4) $$P_{n}={\left|a_{opt,n}\right|}^{2},\phi_{n}=arg\left(a_{opt,n}\right)$$

The test receiving ports represent the prescribed radiation directions and are placed at equal distances from the transmitting-array phase center. Their transmission responses describe the coupling from the transmitting elements toward the selected directions. Under reciprocal conditions, this correspondence follows the transmit–receive relation of the electromagnetic fields [29].

In the WMMPTE formulation [24], the transmitting and test receiving antennas form a multiport network. The transmitting-to-receiving transmission matrix and receiving-port weights give a weighted-power objective whose maximization leads to the eigenvalue problem for the complex transmitting-port excitations. Thus, the receiving-port positions determine the beam directions, and their weights regulate the relative contributions of those directions.

## 3. Nine-Beam Antenna Model

**Figure 1:**
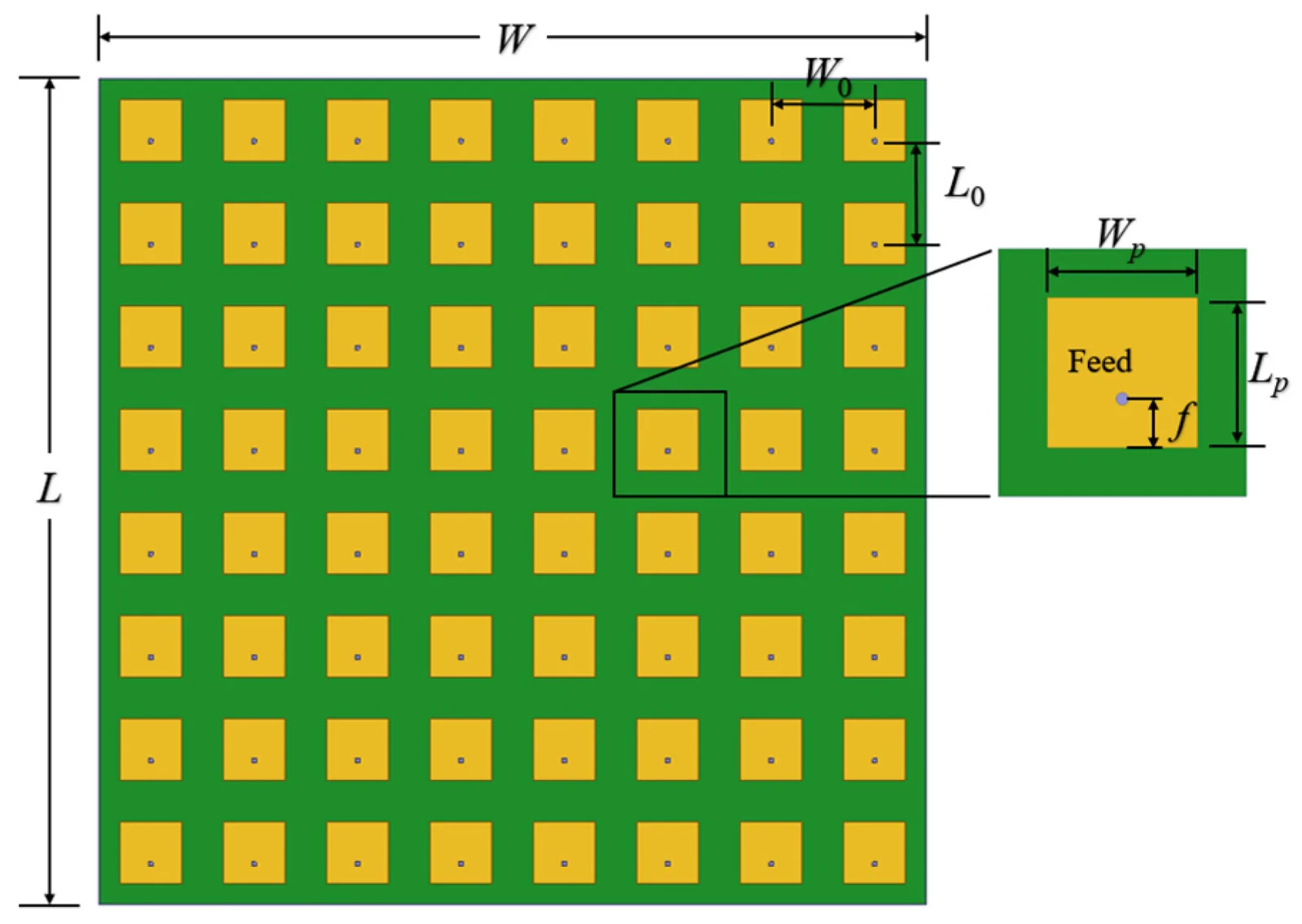
The 8 × 8 Tx array model.

A microstrip antenna element operating at 5.8 GHz is used as the basic radiating element. The Tx array is arranged in an $N_{x}\times N_{y}$ configuration, as shown in Figure 1, and contains 64 Tx ports. The array aperture, element spacing, and microstrip array modeling method are determined with reference to classical antenna array and phased-array design theory [30,31]. The main aperture parameters are listed in Table 1.

The 5.8 GHz transmitting array is modeled in HFSS using a 26 mm × 26 mm periodic microstrip unit cell. The square patch width is 18 mm. The Rogers RT/duroid 5880 substrate is 1.0 mm thick, with a relative permittivity of 2.2 and a loss tangent of 0.0009. The probe feed is offset by 2.75 mm, has a radius of 0.65 mm and a modeled extension of 5 mm; the Teflon feed dielectric has a relative permittivity of 2.1 and a loss tangent of 0.001. Metal surfaces are modeled as PEC, and the element is excited through a 50 Ω lumped port. The lateral boundaries are periodic lattice pairs, with a radiation boundary in the exterior region. The adaptive driven solution uses 5.8 GHz, at most 20 passes, a maximum normalized-power change of 0.02, and a regular 8 × 8 array definition for the 64 active elements at a 26 mm period.

The nine-beam model consists of one central beam and eight off-axis beams. The central beam is along the array normal, i.e., the +z direction. The other eight beams are located on the same off-axis conical surface and are uniformly distributed in azimuth. In the reference case, the off-axis angle is set to 45° to verify equal-gain nine-beam formation and weighted gain control. In the off-axis-angle scanning cases, only the off-axis angle of the conical surface is changed, while the azimuthal angles remain uniformly distributed at 0°, 45°, 90°, 135°, 180°, 225°, 270°, and 315°. Therefore, each scanning state still contains one central beam and eight off-axis beams. Let θ denote the off-axis angle relative to the +z axis, and φ denote the azimuth angle. Then the nine reference target directions can be written as

(5) $$\left\{\begin{array}{c} B_{0}:\theta_{0}=0^{\circ },\varphi_{0}=0^{\circ }\\ \;\;\;B_{i}:\theta_{i}=45^{\circ },\varphi_{i}=\left(i-1\right)\times 45^{\circ },i=1,2,\ldots ,8 \end{array}\right.$$

**Figure 2:**
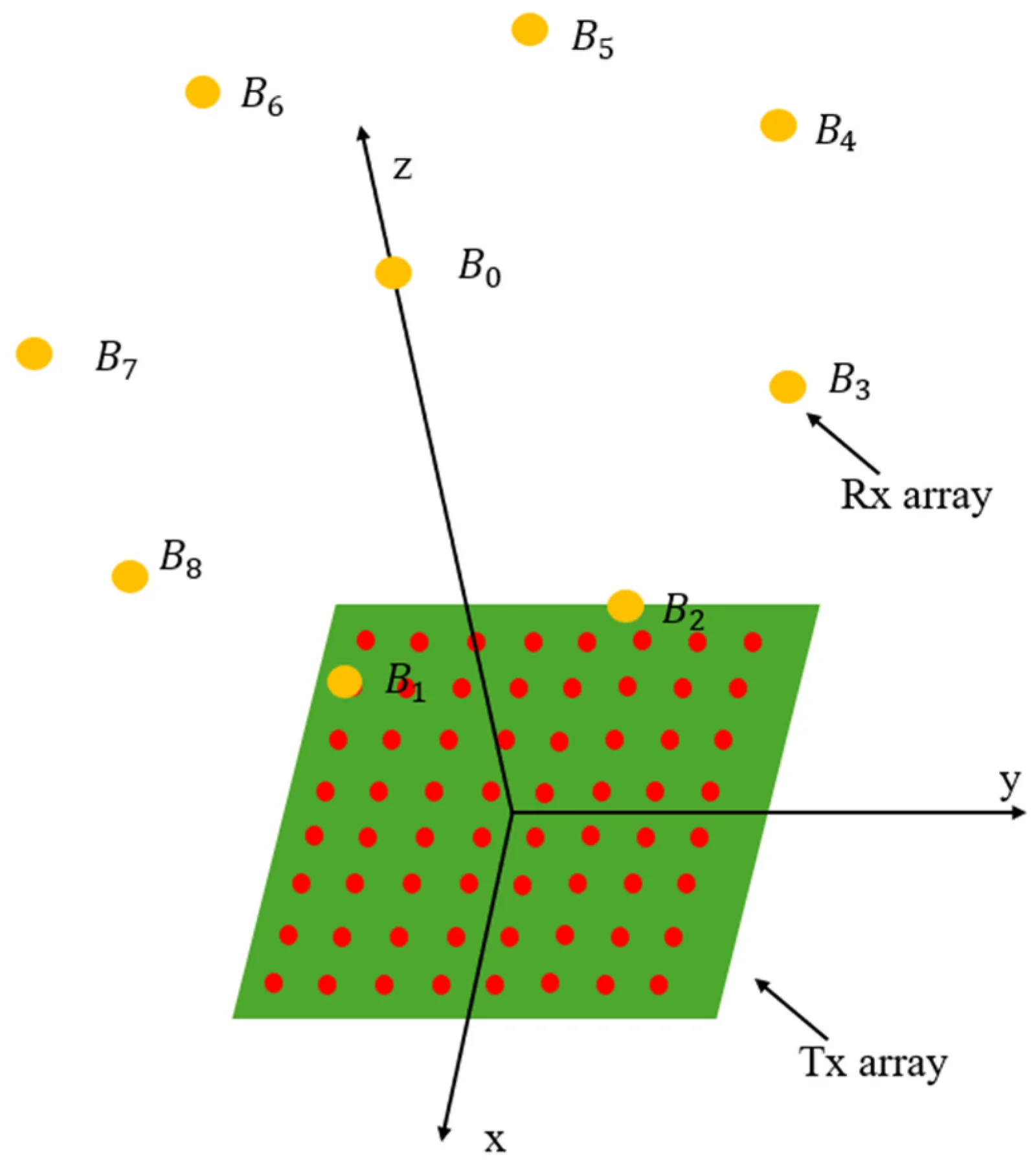
Tx and Rx arrays model.

In the simulation model, one Rx antenna is placed in each target direction, and the distances from the nine Rx antennas to the phase center of the Tx array are kept the same. This arrangement reduces the propagation-loss difference caused by different distances and makes the WMMPTE solution mainly reflect the relationship between the target directions and the radiation capability of the array [31]. The whole Tx-Rx arrays model is shown in Figure 2.

## 4. Results and Analysis

### 4.1. Reflection Coefficients at Representative Array Locations

**Figure 3:**
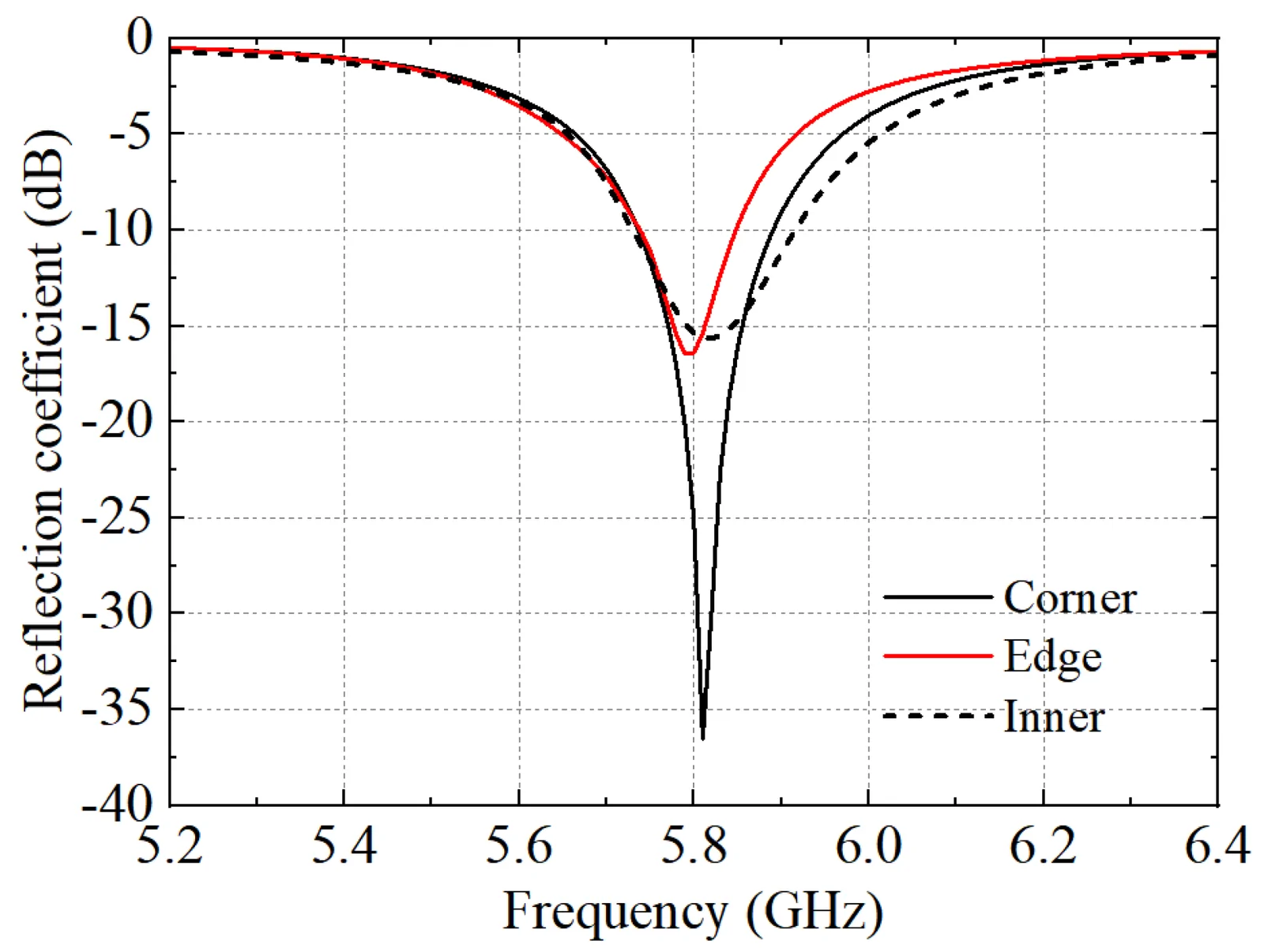
Simulated reflection coefficients of the corner, edge, and inner elements in the 8 × 8 array over 5.2–6.4 GHz.

**Figure 4:**
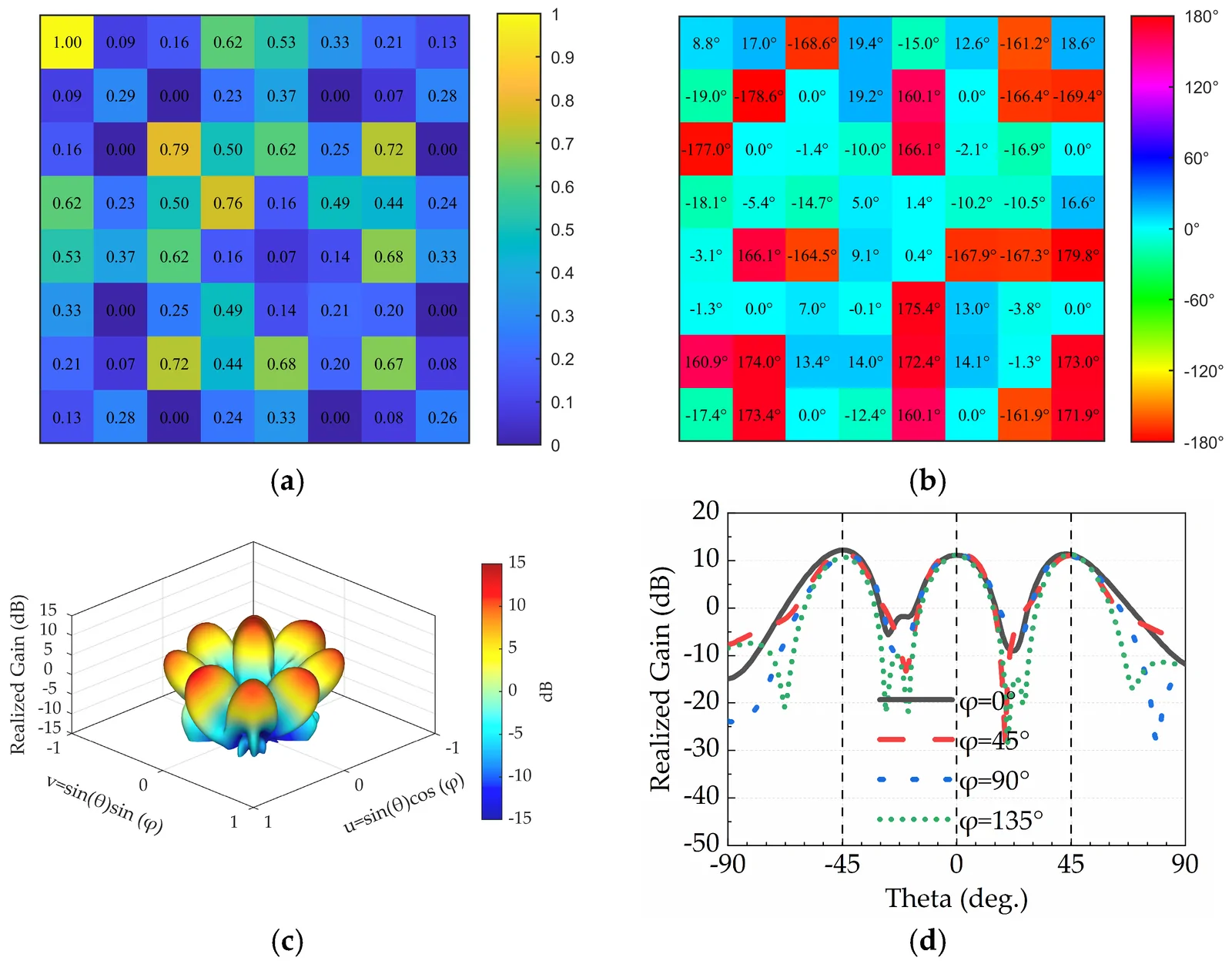
The results of a nine-beam equal-gain configuration: (**a**) normalized amplitude distribution feeding each array element, (**b**) phase distribution of each element, (**c**) three-dimensional radiation pattern, and (**d**) normalized gain patterns for different cuts.

**Figure 5:**
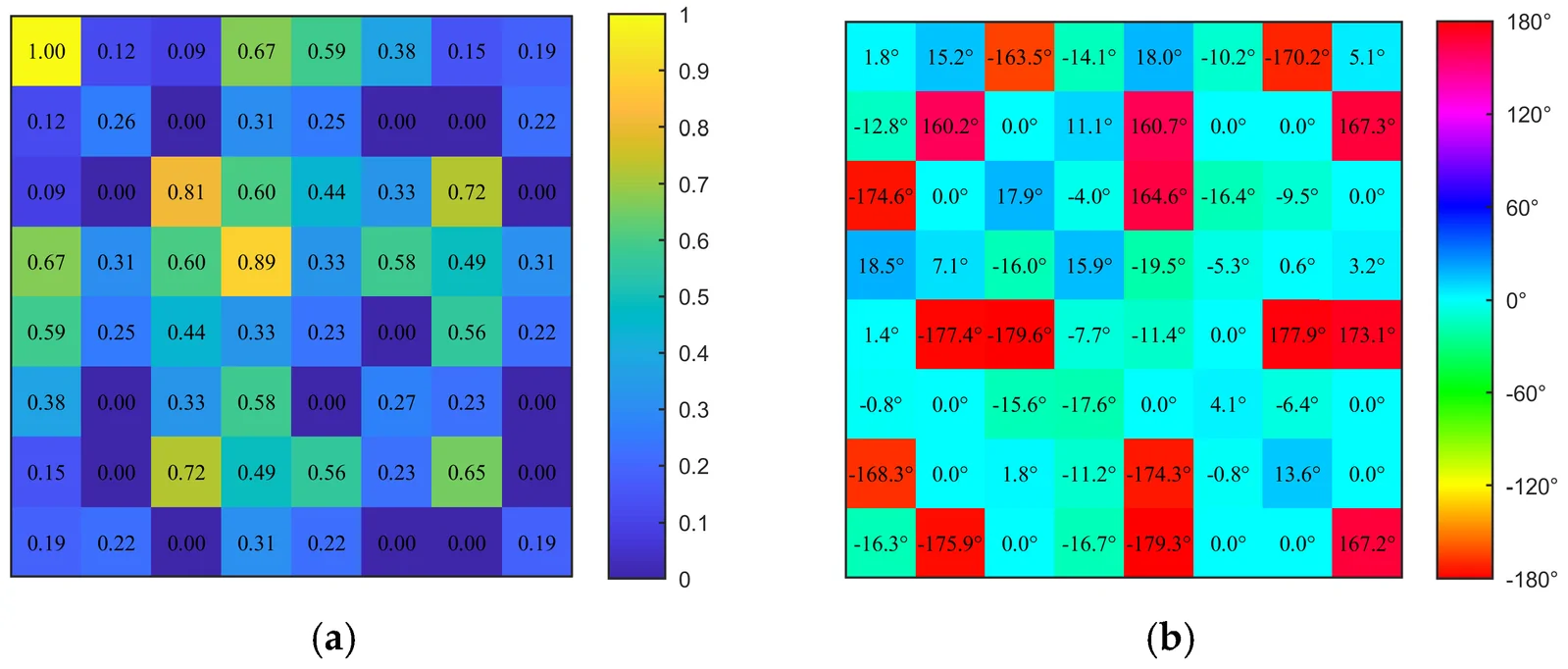

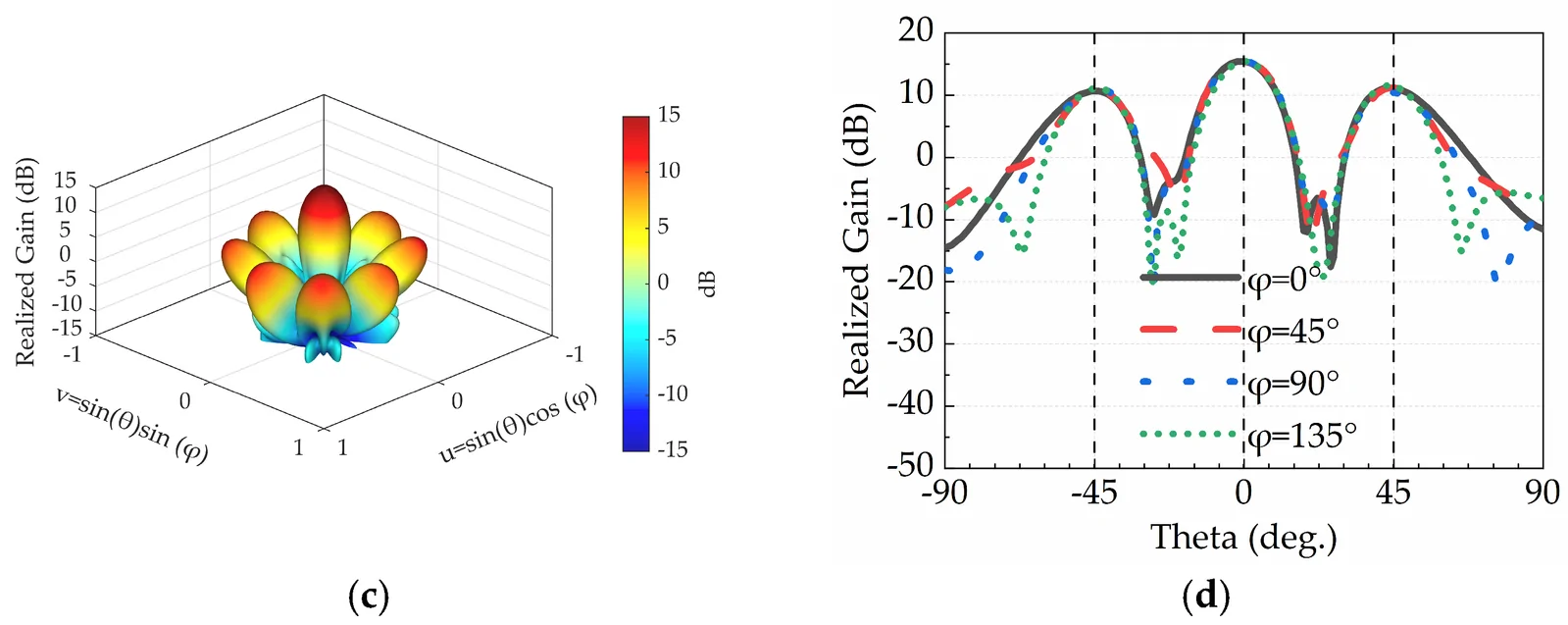
The results where the gain of beam is higher than that of the other beams: (**a**) normalized amplitude distribution feeding each array element, (**b**) phase distribution of each element, (**c**) three-dimensional radiation pattern, and (**d**) normalized gain patterns for different cuts.

**Figure 6:**
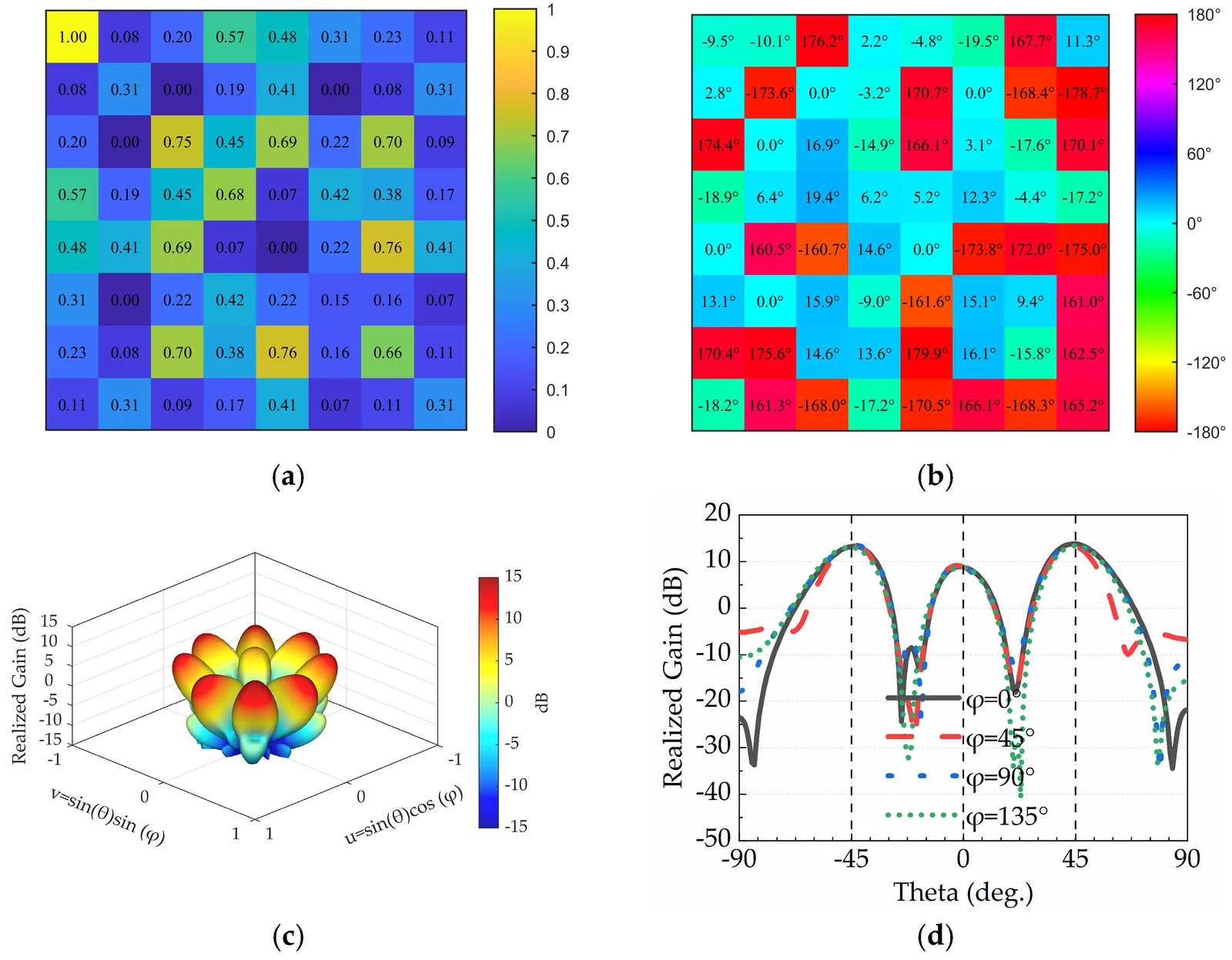
The results where the gain of beam is lower than that of the other beams: (**a**) normalized amplitude distribution feeding each array element, (**b**) phase distribution of each element, (**c**) three-dimensional radiation pattern, and (**d**) normalized gain patterns for different cuts.

**Figure 7:**
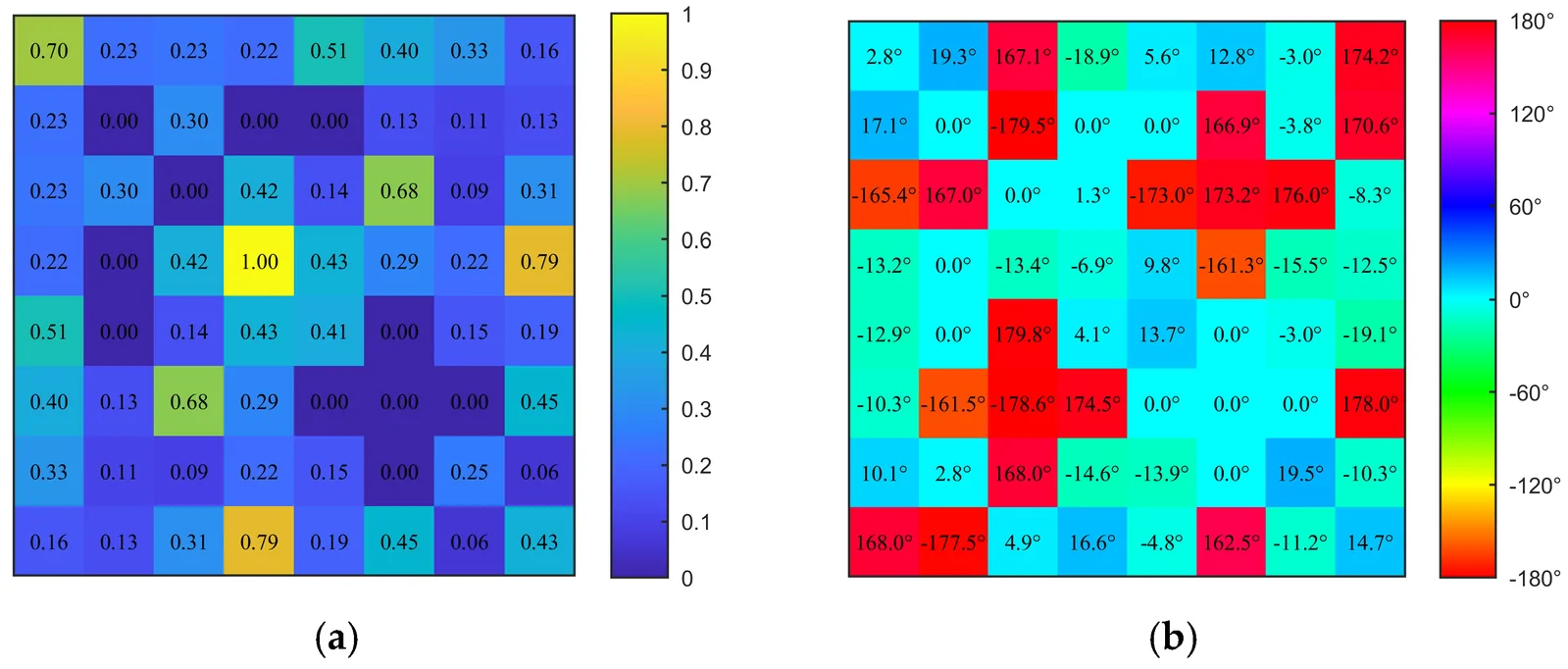

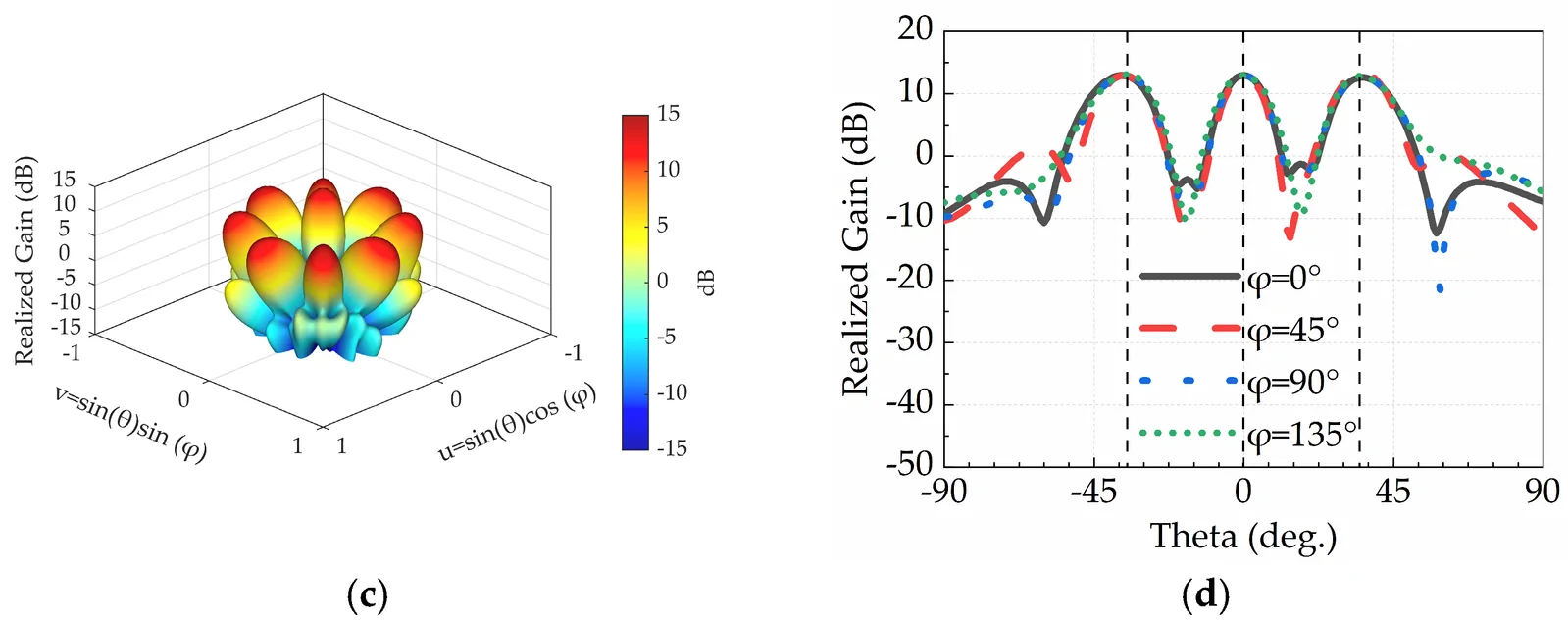
The results of the nine-beam equal-gain configuration at = 35°: (**a**) normalized amplitude distribution feeding each array element, (**b**) phase distribution of each element, (**c**) three-dimensional radiation pattern, and (**d**) normalized gain patterns for different cuts.

**Figure 8:**
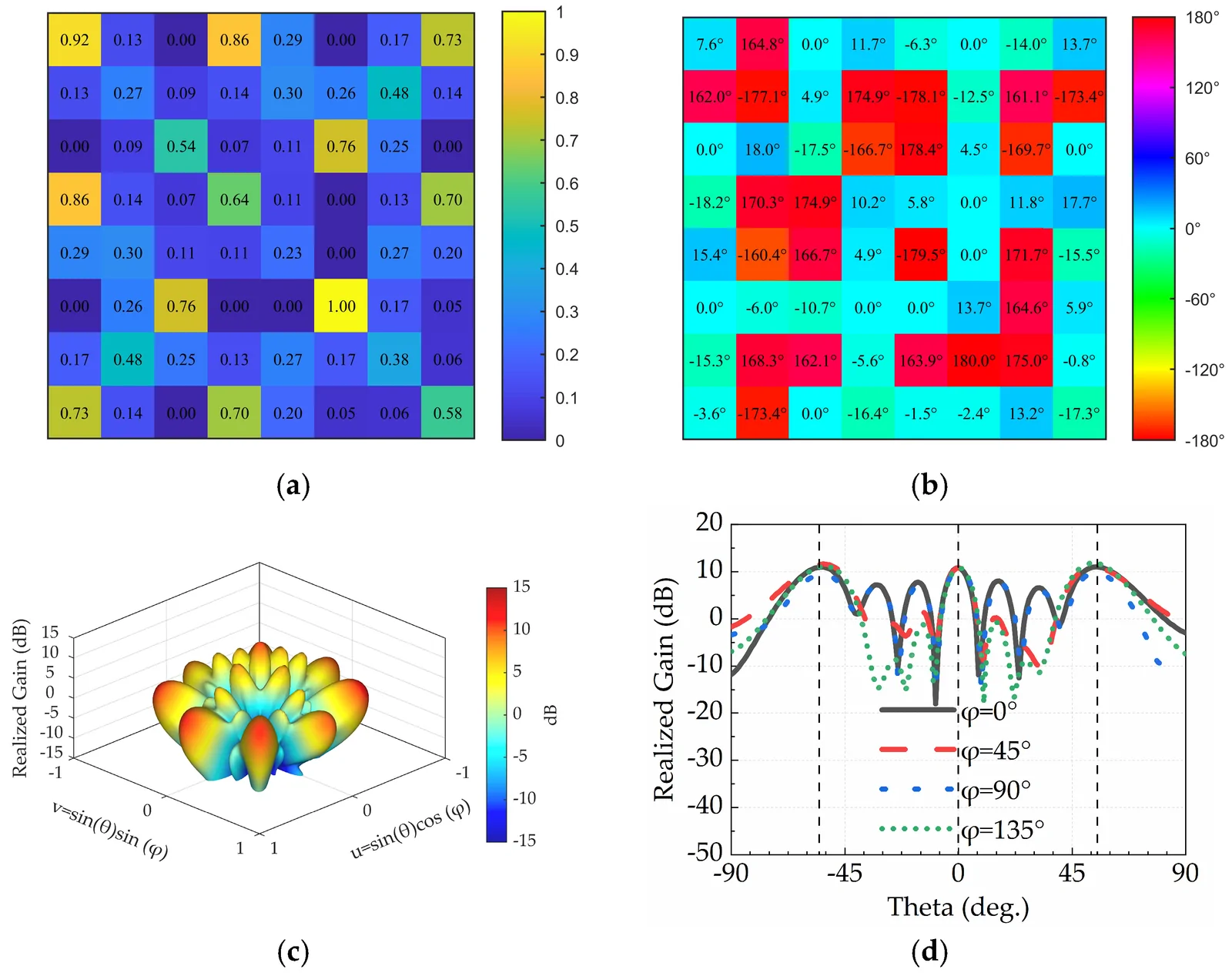
The results of the nine-beam equal-gain configuration at = 55°: (**a**) normalized amplitude distribution feeding each array element, (**b**) phase distribution of each element, (**c**) three-dimensional radiation pattern, and (**d**) normalized gain patterns for different cuts.

Figure 3 compares the simulated reflection coefficients of elements at a corner, an edge, and an inner position of the 8 × 8 array over 5.2–6.4 GHz. The resonance minima occur at 5.81, 5.79, and 5.82 GHz, respectively. At 5.8 GHz, the corresponding reflection coefficients are −25.13, −16.41, and −15.38 dB, indicating good matching at the operating frequency. The full-wave electromagnetic array responses, including the modeled element interactions, are used with the WMMPTE excitations to obtain the nine-beam patterns shown in Figure 4, Figure 5, Figure 6, Figure 7 and Figure 8.

### 4.2. Unweighted Nine-Beam Formation

To verify the formation of the prescribed nine beams, the equal-weight case is considered first. The Tx-side weighting matrix is set as [W]=[I], indicating that the nine prescribed target directions are assigned equal weights in the objective function. Therefore, no target direction is preferentially emphasized during the optimization, and the resulting excitation can be regarded as the reference case for balanced nine-beam synthesis. By solving (3), the 64-port complex excitation vector corresponding to the maximum power transmission efficiency is obtained. The calculated excitation coefficients determine the amplitude and phase applied to each transmitting element and are subsequently used to excite.

### 4.3. Weighted Gain Control of the Nine Beams

This subsection examines whether the relative gains of the nine beams can be adjusted through the receiving-port weights while their prescribed directions remain essentially unchanged. Only the broadside Rx-port weight is varied. Increasing this weight gives the broadside direction a larger contribution to the objective function, whereas reducing it shifts the weighting toward the off-axis directions. Figure 4 and Figure 5 present these two cases.

Figure 5 shows the result obtained with an increased broadside Rx-port weight. Compared with the equal-weight case, the broadside gain increases and the gains of the surrounding off-axis beams decrease. The larger weight assigned to the central port causes the WMMPTE solution to allocate more of the available excitation capability to the broadside direction. The off-axis main lobes remain distinct, and their pointing directions change little.

Figure 6 shows the complementary case with a reduced broadside Rx-port weight. The central beam becomes weaker, while the off-axis beams carry more of the radiation toward the surrounding directions.

The two weighting cases show that the relative gain of the central and off-axis beams can be adjusted through the diagonal entries of the weighting matrix while the prescribed beam geometry is retained. The weighting matrix therefore provides a practical variable for selecting the relative emphasis placed on broadside and off-axis coverage.

### 4.4. Off-Axis-Angle Scanning Results

The beam-steering capability of the algorithm is further examined through simulations in this subsection. The reference case uses as $\theta_{i}$ = 45° the off-axis angle. To examine other coverage states, the off-axis angle of the conical surface containing the eight receiving ports is changed, and the WMMPTE solution is recalculated. The cases $\theta_{i}$ = 35°, 45°, and 55° are considered. Each case retains one broadside beam along +z and eight azimuthally uniform off-axis beams; only the angle between the off-axis beams and the array normal changes.

Figure 7 shows the nine-beam pattern at a smaller off-axis angle. As the corresponding Rx ports move closer to the array normal, the eight off-axis peaks move toward broadside, while the central beam remains near = 0°. The surrounding beams retain their azimuthal arrangement. Redefining the Rx-port locations and resolving the Tx excitation vector changes the coverage state while retaining the nine-beam configuration.

Figure 8 shows the nine-beam pattern at a larger off-axis angle. Relative to Figure 7, the off-axis main lobes are farther apart and the transition-region nulls shift. At larger scan angles, element-pattern roll-off, projected-aperture reduction, and inter-beam coupling have a stronger effect on the finite-aperture pattern. These effects set practical constraints on the scanning angle, allowable sidelobe level, and feeding dynamic range.

## 5. Discussion and Comparison

For clarity, the simulation results shown in Figure 4, Figure 5, Figure 6, Figure 7 and Figure 8 are denoted as case 1 to case 5, respectively. Case 1 is the equal-gain nine-beam reference case at an off-axis angle of 45°. Case 2 and case 3 are used to evaluate the gain-redistribution capability by increasing and decreasing the weight of the central beam, respectively. Case 4 and case 5 are used to evaluate the off-axis-angle reconfiguration capability under equal-gain weighting.

In Table 2, ΔG is defined as the gain difference between the central beam $B_{0}$ and the average gain of the eight off-axis beams. A positive value means that the central beam is stronger than the off-axis beams, whereas a negative value indicates that the central beam is weaker. The comparison among case 1, case 2, and case 3 shows that, at the same off-axis angle of 45°, the proposed WMMPTE method can redistribute the relative gain between the central beam and the off-axis beams by changing the receiving-port weights.

Table 2 lists the prescribed and realized ΔG values for Cases 1–3. The expected values are 0, +5, and −5 dB, whereas the full-wave realized-gain results are 0.06, +5.08, and −5.03 dB. The corresponding absolute deviations are 0.06, 0.08, and 0.03 dB, respectively. For the same cases, the gain fluctuations among $B_{1}$–$B_{8}$ are 0.8, 0.9, and 0.7 dB, respectively, while the maximum gains of the complete nine-beam patterns are 12.0, 15.5, and 13.8 dBi.

With the receiving-port order $B_{0}$–$B_{8}$, the weights of $B_{1}$–$B_{8}$ remain 1, while the $B_{0}$ weights are 1, 1.171, and 0.932 for Cases 1–3, respectively. The corresponding diagonal matrices are W=diag(1,1,1,1,1,1,1,1,1), W=diag(1.171,1,1,1,1,1,1,1,1), and W=diag(0.932,1,1,1,1,1,1,1,1). The unequal weight increments required for successive 5 dB allocations show that the relationship between the central receiving-port weight and ΔG is nonlinear. Intermediate allocations are obtained by adjusting the central weight and resolving the eigenvalue problem.

Beyond the three representative cases in Table 2, additional HFSS full-wave calculations were carried out for prescribed ΔG values of 0, 2, 5, 8, and 10 dB using the same nine-beam geometry. Across the four examined azimuthal cuts, the relative separation between the broadside and surrounding main-lobe levels increases progressively with ΔG, while the surrounding main lobes remain near their prescribed directions. The principal nine-beam structure is retained throughout the investigated 0–10 dB range.

Table 3 compares the nine-beam results under different off-axis angles. In all three cases, the array maintains one central beam and eight off-axis beams. When the off-axis receiving ports are moved from 35° to 55°, the corresponding off-axis beams move with the target directions after the array excitation is recalculated. This result shows that the proposed method can reconfigure the spatial coverage angle while maintaining the basic nine-beam configuration.

Considering the five configurations together, the SLLs of Cases 1–5 are −10.4, −9.8, −10.1, −10.7, and −8.7 dB, respectively, giving a maximum variation of 2.0 dB. Cases 1–3 correspond to different gain-allocation states, whereas Cases 4 and 5 correspond to different spatial-coverage states. The changes in array excitation therefore affect the sidelobes and nulls in addition to the main-beam gains and directions. For additional SLL control, field responses in selected sidelobe directions can be included in the weighted synthesis objective with corresponding suppression weights.

The excitation dynamic-range ratios (DRRs) for Cases 1–5 are 49.1, 53.6, 51.1, 50.2, and 58.4 dB, respectively, calculated from the largest and smallest nonzero magnitudes of the 64 transmitting-port excitations. These amplitude spans place corresponding requirements on the feeding power ratios and port amplitudes. For a fixed gain-allocation state, unequal T-junction power dividers can set the branch power ratios, and transmission-line lengths can provide the required phase differences [24]; line impedances, matching, and feed loss also need to be considered for the large DRRs.

A multistage unequal power-divider network may be considered to accommodate the large excitation-amplitude range across the array ports. Such power division places demanding requirements on branch impedance transformation, matching, and amplitude accuracy, particularly for weakly excited ports. Feed loss and transmission-line fabrication tolerances may further affect the realized amplitude and phase distributions.

To compare the characteristics of different multibeam implementation approaches, Table 4 summarizes representative schemes based on fixed beamforming networks, genetic algorithms (GA), convex optimization (CO), particle swarm optimization (PSO), and simulated annealing (SA) [19,20,21,22,23]. Since these studies differ in operating frequency, array configuration, and design objective, the comparison mainly focuses on the number of simultaneously generated beams, whether iterative optimization is required, whether relative gain control among the beams is supported, and the maximum beam direction.

Reference [19] uses a Butler matrix to generate four discrete beams without numerical iteration. Its beam count and amplitude-phase relationships follow the hardware network, which limits inter-beam gain control. Reference [20] uses a genetic algorithm to synthesize seven target beams with a 64-element array, at the cost of multigeneration iterative searching. In [21], convex optimization is applied to a 64-element array over multiple scan states and can constrain the scan range and sidelobe level. The reported multiple beams, however, are distinct scan states rather than concurrent main beams from one excitation set, and the solution remains iterative. Reference [22] uses PSO to search the element phases of a single-feed reflectarray. Its Ka-band quad-beam prototype has different pointing directions and gain levels, showing that stochastic optimization can redistribute gain among beams. Reference [23] uses a time-modulated array and simulated annealing to optimize the switch-on duration and instant of each element for multibeam synthesis. This procedure is iterative and does not set relative beam gain as an independent design objective.

Using the largest absolute beam direction reported or demonstrated in each study, the maximum directions in [19,20,21,22,23] are 43°, 42.43°, 51.8°, 45°, and 45°, respectively. The present array is evaluated up to 55°, while simultaneously maintaining nine beams. These values are summarized in the final column of Table 4 together with the beam number, iterative-optimization requirement, and relative-gain-control capability.

In this work, a 64-element array is used to simultaneously generate nine target beams with a single set of array excitations. Compared with fixed beamforming networks, the relative beam gains are not predetermined by the hardware network. Compared with genetic-algorithm and convex-optimization-based approaches, once the transmission matrix and weighting matrix are specified, the complex array excitations can be obtained directly through eigenvalue decomposition without multistep iterative searching. Furthermore, by adjusting the receiving-port weights, the relative gains among different beams can be controlled while their target directions remain essentially unchanged.

After the transmission parameters have been obtained from full-wave electromagnetic analysis, the 64-element array excitations follow from a 64 × 64 eigenvalue problem. In MATLAB R2020b, matrix construction takes approximately 0.02 ms on average and the eigenvalue solution approximately 0.56 ms. For fixed target directions, changing the receiving-port weights only requires resolving the eigenvalue problem with the corresponding weights, without iterative searching over candidate excitations.

In the present gain-allocation configuration, the eight off-axis receiving-port weights remain fixed at 1, while only the central-port weight is adjusted. The results in Table 2 show that increasing this weight raises the broadside-beam gain relative to the surrounding beams, whereas decreasing it lowers the relative gain. Although the relationship between the central-port weight and the realized gain difference is nonlinear, this trend allows a suitable weight ratio to be determined through a small number of trial adjustments within the investigated range. The corresponding array excitation is obtained directly from the eigenvalue solution for each selected weighting matrix.

Sensitivity to excitation errors was evaluated for the +5 dB gain-allocation case under four representative amplitude and phase perturbation conditions: ±1% amplitude with ±1° phase, ±5% amplitude with ±1° phase, ±1% amplitude with ±5° phase, and ±5% amplitude with ±5° phase. The corresponding realized |ΔG| values are 5.03, 5.28, 5.21, and 5.33 dB, respectively, so the largest deviation from the nominal 5 dB gain difference is 0.33 dB. The SLLs over these four cases range from −9.8 to −8.3 dB, and the maximum beam-pointing deviation does not exceed 1.6°. Feed-line losses and fabrication tolerances can affect the amplitudes and phases delivered to the antenna ports.

## 6. Conclusions

A nine-beam array is synthesized with the WMMPTE formulation. Moving the receiving-port locations shifts the beam-coverage directions, and retuning the port weights redistributes the relative gains while the prescribed directions change little. At an off-axis angle of 45°, the gain difference between the central beam and the average of the eight off-axis beams spans −5 to +5 dB. Sweeping the off-axis angle from 35° to 55° preserves the nine-beam configuration, with the maximum gain ranging from 11.8 to 13.3 dBi. Pointing and gain map onto the port locations and port weights, respectively, and the array excitation follows from the same multiport-network model. A fixed feeding network sets the gain ratio once; iterative synthesis reaches it only by searching. Here, both follow from one choice of port locations and weights, which makes the link between the design variables and the beam characteristics direct. This property is useful for multibeam arrays requiring unequal gain allocation among predefined coverage directions.

## Figures and Tables

**Table 1:** Array parameters.

Parameter	Value (mm)	Parameter	Value (mm)
*W*_0_	26	*L*_0_	26
*W*	208	*L*	208
*W_P_*	18	*L_P_*	18
*f*	5		

**Table 2:** Gain-redistribution results at the same off-axis angle.

Simulation Case	Off-Axis Angle (°)	Number of Beams	Max Gain (dBi)	Expected/Realized ΔG (dB)
case 1	45	9	12.0	0/0.06
case 2	45	9	15.5	+5/+5.08
case 3	45	9	13.8	−5/−5.03

**Table 3:** Off-axis-angle reconfiguration results under equal-gain weighting.

Simulation Case	Off-Axis Angle (°)	Number of Beams	Max Gain (dBi)	ΔG (dB)
case 1	45	9	12.0	0
case 4	35	9	13.3	0
case 5	55	9	11.8	0

**Table 4:** Performance comparison with published multibeam scanning arrays.

Reference	Method	No. of Beams	Iterative Optimization	Relative Gain Control	Maximum Beam Direction (°)
[19]	Butler	4 (switched)	×	×	43
[20]	GA	7	√	√	42.43
[21]	CO	1 per scan state	√	√	51.8
[22]	PSO	4	√	√	45
[23]	SA	3	√	×	45
This work	WMMPTE	9 concurrent	×	√	55

Note: √ indicates yes; × indicates no.

## Data Availability

Data are contained within the article.
